# Integrated Stress Response and Decreased ECM in Cultured Stromal Cells From Keratoconus Corneas

**DOI:** 10.1167/iovs.18-24367

**Published:** 2018-06

**Authors:** James W. Foster, Vishal Shinde, Uri S. Soiberman, Gajanan Sathe, Sheng Liu, Julius Wan, Jiang Qian, Yassine Dauoud, Akhilesh Pandey, Albert S. Jun, Shukti Chakravarti

**Affiliations:** 1 Wilmer Eye Institute, Johns Hopkins University School of Medicine, Baltimore, Maryland, United States; 2 Department of Ophthalmology, NYU Langone Health, New York, New York, United States; 3 Manipal Academy of Higher Education, Karnataka, India; 4 Department of Medical and Molecular Genetics, Indiana University School of Medicine, Indianapolis, Indiana, United States; 5 Department of Biological Chemistry, Johns Hopkins University School of Medicine, Baltimore, Maryland, United States

**Keywords:** keratoconus, collagen, extracellular matrix, cellular stress

## Abstract

**Purpose:**

Keratoconus (KC) is a multifactorial disease where progressive thinning and weakening of the cornea leads to loss of visual acuity. Although the underlying etiology is poorly understood, a major endpoint is a dysfunctional stromal connective tissue matrix. Using multiple individual KC corneas, we determined that matrix production by keratocytes is severely impeded due to an altered stress response program.

**Methods:**

KC and donor (DN) stromal keratocytes were cultured in low glucose serum-free medium containing insulin, selenium and transferrin. Fibronectin, collagens and proteins related to their chaperone, processing and export, matrix metalloproteinase, and stress response related proteins were investigated by immunoblotting, immunocytochemistry, hydroxyproline quantification, and gelatin zymography. Multiplexed mass spectrometry was used for global proteomic profiling of 5 individual DN and KC cell culture. Transcription of selected proteins was assayed by qPCR.

**Results:**

DN and KC cells showed comparable survival and growth. However, immunoblotting of selected ECM proteins and global proteomics showed decreased fibronectin, collagens, PCOLCE, ADAMTS2, BMP1, HSP47, other structural and cytoskeletal proteins in KC. Phosphorylated (p) eIF2α, a translation regulator and its target, ATF4 were increased in KC cultured cells and corneal sections.

**Conclusions:**

The profound decrease in structural proteins in cultured KC cells and increase in the p-eIF2α, and ATF4, suggest a stress related blockade in structural proteins not immediately needed for cell survival. Therefore, this cell culture system reveals an intrinsic aggravated stress response with consequent decrease in ECM proteins as potential pathogenic underpinnings in KC.

Keratoconus (KC) is a multifactorial disease where the cornea becomes weak and progressively thin.^1–3^ Affecting 1 in 2000 individuals, it is a common cause of blindness and often results in the need for cornea transplantation. While some cases are syndromic, associated with other connective tissue disorders, the vast majority is non-syndromic and sporadic.^4,5^ Nonsyndromic keratoconus is classically described as a bilateral non-inflammatory corneal ectasia and thinning resulting in abnormal topography, astigmatism, and myopia as some of the earliest features.^6^ The cornea is the outermost protective barrier that combines transparency with refractive power for optimal vision.^7,8^ This is largely achieved by the stroma, a highly organized extracellular matrix (ECM) of stacked collagen fibrils interspersed with flattened keratocytes, the cells responsible for producing and maintaining the ECM.^9^ The predominant, strength-bearing collagen is type I, making up ∼60% of all collagen in the corneal stroma. Collagen type V, described as an initiator of type I collagen fibril formation at the cell surface, makes up a further 10% to 20% of the stromal total collagen.^10,11^

Previous investigations of keratoconus, both in vitro and ex vivo, have identified collagen extractability as being unchanged,^12^ suggesting that assembly itself is not at fault. Similarly, metalloproteinases, lysosomal enzymes, and tissue inhibitor of matrix metalloproteinases were reported as increased or unchanged.^13,14^ In vitro and in vivo observations also cite keratocyte loss, mitochondrial DNA damage, and increased expression of matrix degradation enzymes as hallmarks of KC.^15,16^ Mass spectrometric analyses of KC and donor corneal tissue extracts have also identified significant decreases in collagen types I and III and other stromal ECM proteins.^17^

We hypothesized that an underlying defect in the keratoconus stromal ECM is functional impairment in keratocytes, and that this could be detected and further investigated in cell culture. Our results show a decrease in collagens and additional matrix modifying proteins in KC keratocyte cultures suggesting translational inhibition initiated by integrated stress response signals.^18–20^ Thus, decreased ECM in KC may in part be due to a heightened stress response in stromal keratocytes.

## Methods

### Ethics Statement

All human tissue was obtained under established guidelines related to informed consent for research use of human donor and patient corneas and adhering to the tenets of the Declaration of Helsinki. Informed consent was obtained from keratoconus patients using a protocol approved by the Johns Hopkins Medicine IRB and entitled “Phenotypic and Genotypic Analysis of Keratoconus” (NA_00006544).

### Cell Culture

Human stromal cells were extracted as described previously^21,22^ from cadaverous corneal donors (Lions Eye Bank, Baltimore, and Lions Eye Institute for transplant and research, Tampa), or obtained with consent from surgical samples after corneal transplantation in confirmed keratoconus patients (Wilmer Eye Institute, Baltimore). Expanded corneal fibroblasts were seeded into plates at a density of 1 × 10^4^ cells/cm^2^ in FGM and allowed to adhere overnight. The next day the cells were washed with PBS and replaced with low-glucose serum-free (LGSF) DMEM supplemented with 1% insulin, transferrin, selenium (I3146; Sigma-Aldrich Corp., St. Louis, MO, USA) 1mM phosphoascorbic acid and 1% antibiotic/antimycotic solution to revert the cells to a keratocyte-like phenotype.^21–23^ The media was replaced after 24 hours and then subsequently every 2 to 3 days.

### Cell Survival Assay

The alamar blue/resazurin (#R7017; Sigma-Aldrich Corp.) reduction assay was used to quantify total cell numbers. A working concentration of 55 μM resazurin was prepared in LGSF and at each media change 500 μL was applied to the well and incubated at 37°C for 90 minutes. Fluorescence was then measured on a fluorimeter with 540 nm excitation and 590 nm emission, each well was read in duplicate and cell number derived from standard curves of known cell numbers. Cell density was assayed using the live/dead assay (#04511; Sigma-Aldrich Corp.). Wide field images were then taken on an axio-observer (Zeiss; Zeiss GmbH, Oberkochen, Germany) with a ×10 objective, images were taken using commercial software (Cellsense; Zeiss GmbH) with two images taken per well. Image quantification was undertaken using ImageJ software (http://imagej.nih.gov/ij/; provided in the public domain by the National Institutes of Health, Bethesda, MD, USA).

### Sirius Red

Collagen production was assessed using a modified sirius red staining method.^24^ Briefly, cells were maintained as above and on day 14 were washed with PBS and fixed for 30 minutes in 4% PFA in PBS, and then stained with a 0.1% solution of picrosirius red (#STPSRPT; American Mastertech, Lodi, CA, USA), which was applied to the cell layer for 30 minutes with gentle rocking. The stain was then aspirated, the cell layer washed three times with distilled water for 15 minutes with gentle rocking. Images were then acquired using cell-sense software (Zeiss GmbH) on an axio-imager (Zeiss GmbH) with a ×20 objective.

### Hydroxyproline Assay

Hydroxyproline was assayed using the hydroxyproline kit (MAK008; Sigma-Aldrich Corp.). Total cell layer, total media, or media and cell layer from each biological repeat was lyophilized and then resuspended in 100 μL of distilled water and 50 μL were carried forward to quantification as per manufacturer's instructions, all samples were assayed in duplicate.

### Fluorescent Imaging

Cells were grown on glass coverslips, fixed in 4% paraformaldehyde in PBS for 15 minutes, the fixative removed and the cells permeabilized with 0.1% Triton X-100 for 5 minutes. The cells were then blocked in 3% bovine serum albumin (Sigma-Aldrich Corp.) and 2% normal goat/donkey serum (corresponding to the species that the secondary antibody to be used was raised in) for 1 hour at room temperature. All antibodies (Supplementary Table S2) were diluted in this blocking buffer and incubated overnight at 4°C. F-actin was labeled with AlexaFluor 568 conjugated phalloidin, diluted 1:200 in wash buffer for 15 minutes. Images were acquired on an axio-observer (Zeiss GmbH) with a ×20 objective, with images captured using commercial software (Zeiss GmbH).

### Western Blotting

Proteins were detected using standard Western blotting techniques. Briefly, cells were solubilized in RIPA buffer with protease inhibitors (#11697498001; Roche, Basel, Switzerland) using a cell scraper. The samples were then sonicated and protein concentration recorded using the BCA assay (Sigma-Aldrich Corp.). For collagen I quantification in cell layer samples, 10μg of total protein per lane was run on 4% to 15% gradient gels (Bio-Rad Laboratories, Hercules, CA, USA). For analyzing media proteins stain-free gradient gels (Bio-Rad Laboratories) were used to quantify total protein loaded after 1 minute of UV activation. Proteins were then transferred to polyvinylidene fluoride (PVDF) membranes using wet transfer apparatus then blocked using 5% non-fat dry milk or bovine serum albumin in PBS for 1 hour at room temperature. Primary antibodies were diluted as appropriate (Supplementary Table S1) in blocking buffer with 0.1% Tween 20 and incubated at 4°C overnight. Membranes were washed 3 × 15 minutes in PBS and 0.1% Tween 20. Horseradish peroxidase–conjugated secondary antibodies were diluted 1:5000 in blocking buffer and incubated for 1 hour at room temperature. Chemiluminescent substrate was next applied to the membrane and gels imaged on a commercial system (Chemidoc M; Bio-Rad Laboratories). Band intensity was plotted relative to the total protein or glyceraldehyde 3-phosphate dehydrogenase (GAPDH) using ImageJ.

### Quantitative PCR

RNA was extracted from cells using a commercial kit (RNEasy; Qiagen, Valencia, CA, USA) and 1 μg of RNA reverse transcribed using a reverse transcription kit (Superscript III, 18080093; Thermo Fisher Scientific, Waltham, MA, USA), per the manufacturer's instructions. A total of 3 ng cDNA was subjected to qPCR using SYBR green reagents (ABI 4309155) on a thermocycler (ABI 7900HT; Thermo Fisher Scientific) using exon-spanning gene-specific primers (Supplementary Table S3). Each test was run in triplicate with relative transcript levels of target genes expressed as 2−ΔcT with POLR2A as the housekeeping control gene.

### Gelatin Zymography

Zymography was carried out as described previously.^25^ Briefly, equal volumes of media were run under nonreducing conditions in a 10% acrylamide SDS PAGE containing 1% gelatin. The gel was renatured in 2.5% Triton X-100 and subsequently developed. Gels were stained with Coomassie blue and imaged using a commercial system (Bio-Rad Chemidoc M; Bio-Rad Laboratories). Bands were next quantified using ImageJ software with the area under the curve plotted.

### Mass Spectrometry

#### Peptide Preparation and Phosphopeptide Enrichment

Protein from 5 DN and 5 KC cell cultures were extracted in 2% SDS in 50 mM TEABC using a high-pressure instrument (Barocycler NEP2320-45k; Pressure BioSciences, South Easton, MA, USA). After cell lysis equal amounts of protein (100 μg) were reduced (5 mM dithiothreitol) and alkylated (IAA 20 mM). The proteins were then precipitated in chilled acetone to remove SDS. After trypsin digestion peptides were labeled with TMT reagents as per the manufacturer's instructions, pooled and subjected to basic reverse phase liquid chromatography in 12 fractions for liquid chromatography–mass spectrometry (MS)/MS.

#### Analysis

Peptides were reconstituted in 0.1 % formic acid and loaded on a 2-cm trap column (Acclaim PepMap 100, C18, 5 μm particle size, 100 μm i.d. 100 Å pore size; Thermo Scientific) with a flow rate 5 μL/minute. The peptides were resolved on a 50-cm analytical column (Acclaim PepMap 100, C18, 3 μm particle size, 75 μm i.d. 100 Å pore size; Thermo Scientific) with a 90-minute gradient from 7% to 40% acetonitrile in 0.1% formic acid with a flow rate of 300 nL/minute. The spray voltage was set to 2.2 kV. The mass spectrometer was operated in data-dependent acquisition mode. A full MS scan (from m/z 350–1,550) was acquired in a mass analyzer (Orbitrap; Thermo Fisher Scientific) with resolution 120,000 at m/z 200 with a maximum AGC target value of 200,000 ions. The data-dependent MS/MS was carried out using a top speed method with a duty cycle of 3 seconds. Singly charge precursor ions were excluded. Precursor ions with charge states 2 through 7 were sequentially isolated to a target value of 50,000 ions and fragmented in the higher-energy collisional dissociation (HCD) cell using 39% normalized collision energy. The maximum ion injection time for MS and MS/MS were set to 50 and 120 ms, respectively. Fragment ion spectra were detected in Orbitrap mass analyzer with a resolution 30,000 at m/z 200. Dynamic exclusion was enabled one event of fragmentation followed by exclusion of the precursor for next 30 seconds within 10 ppm of the selected m/z. For all measurements with the Orbitrap detector, a lock-mass ion from ambient air (m/z 445.120025) was used for internal calibration.

#### Data Treatment and Protein/Peptide Identification

A commercial software suite (Proteome Discoverer, version 2.1; Thermo Scientific, Bremen, Germany) was used for protein identification and quantitation. Mass spectrometry data was searched against Human RefSeq protein database using SEQUEST search algorithms. The search parameters included trypsin as the protease with maximum of 2 missed cleavage allowed; oxidation of methionine and proline was set as dynamic modifications while static modifications included carbamidomethylation at cysteine and TMT modification at N-terminus of the peptide and lysine. Precursor mass tolerance was set to 10 ppm and fragment mass tolerance was set to 0.05 Da. The false discovery rate (FDR) was calculated by carrying out decoy database searches and peptides and protein scoring better than 1% FDR score cut-off were considered for further analysis.

## Results

### KC Cells Have Comparable Survival in LGSF Medium

**Figure 1:**
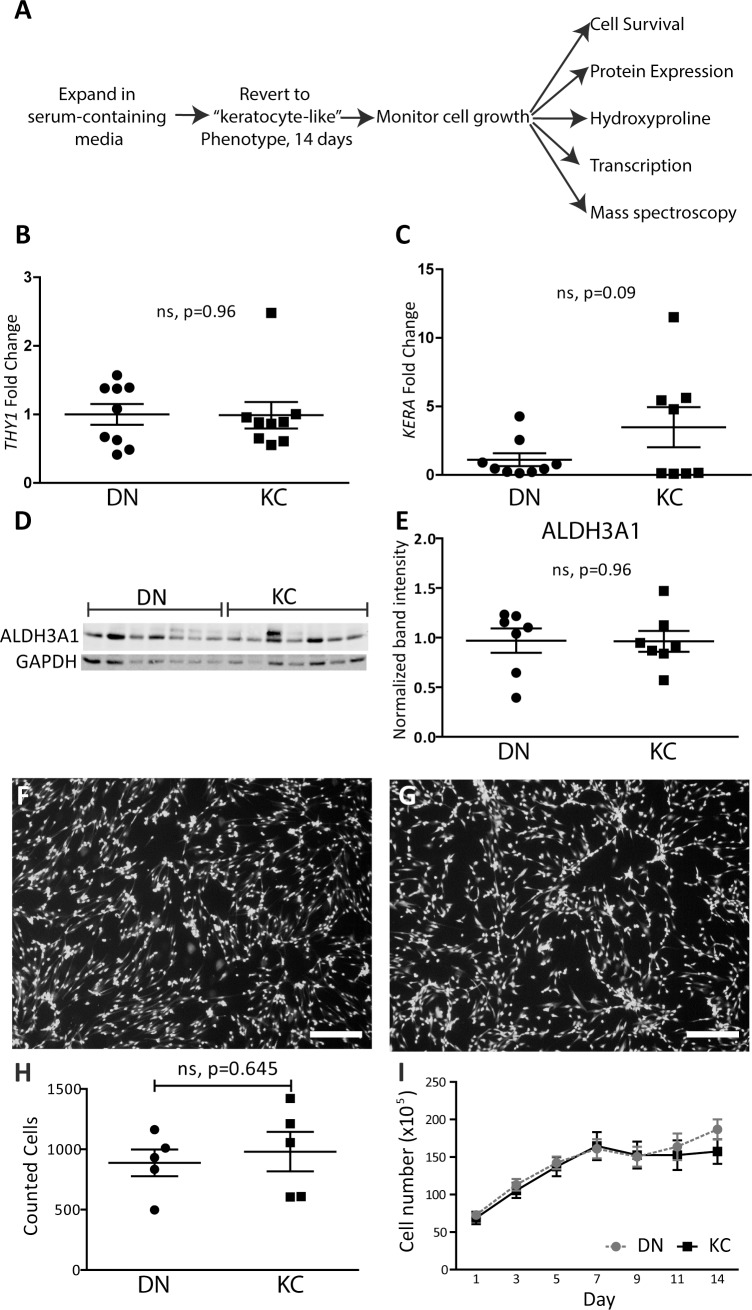
KC cells have comparable survival in LGSF medium. (A) Timeline for inducing the keratocyte phenotype and assessing collagen matrix and cellular phenotypes during 14 days of culture in LGSF. (B) Quantitative PCR fibroblast marker THY1 and (C) of quiescence keratocyte marker KERA; points indicate mean individual expression levels as calculated using 2−ΔΔcT relative to POLR2A, n ≥ 9 biological repeats. (D, E) ALDH3A1 levels in 7 DN and KC keratocyte culture extracts after 14 days; points represent quantification of band intensity normalized to GAPDH. Representative 10X image of live cells (calcein AM) at day 14 in (F) DN and (G) KC cultures, n = 5, scale bar: 200 μm. (H) Quantification of live cells in field of view at day 14, points indicate total live cells in random images from each individual DN or KC culture. (I) Total cell number over duration of cultures calculated from resazurin reduction assay. Values are shown as mean ± SD; statistical significances were calculated using the Student's t-test.

Keratocytes were isolated from 14 keratoconus (KC) and 16 cadaverous donor (DN) corneas (Supplementary Table S1), and maintained as fibroblasts in DMEM:F12 with 5% fetal bovine serum for no more than three passages. KC and DN fibroblasts were cultured in low glucose serum-free media (LGSF) for 2 weeks (Fig. 1A) to induce keratocyte-like features.^22^ In this 14-day LGSF culture system, DN and KC keratocytes displayed: (1) similar morphologies (Supplementary Fig. S1); (2) minimal fibroblastic activation as gauged by low mesenchymal *THY-*1 marker expression^26^ (Fig. 1B); and (3) comparable keratocyte qualities with respect to expression of the keratocyte marker, keratocan (Fig. 1C)^27^ and ALDH3A1, a “corneal crystallin,”^28^ (Figs. 1D, 1E; Supplementary Fig. S2). A count of live, fluorescent Calcein-AM positive cells on day 14 of culture showed similar live cell count in both groups (Figs. 1F–H). Cell numbers monitored every 48 hours through resazurin reduction were also comparable (Fig. 1I).

### ECM Collagens and Fibronectin Decreased in KC Keratocyte Cultures

**Figure 2:**
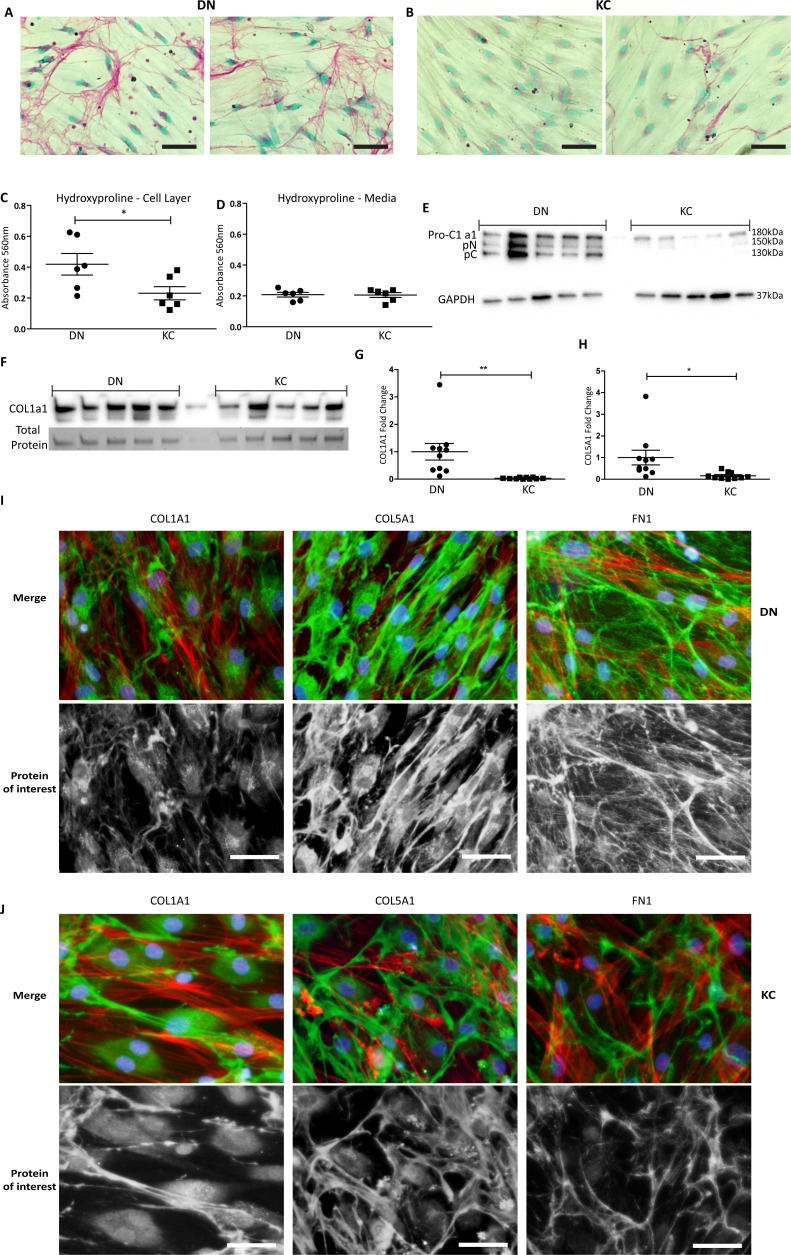
Decreased ECM collagens and fibronectin in KC keratocyte cultures. Representative images of Sirius red and fast green stained cultures (20x) in (A) 2 separate DN and (B) 2 separate KC cultures, scale bar: 50 μm. (C) Cell associated hydroxyproline quantification for total collagenous protein, (D) media associated hydroxyproline. Each point representing means of three measurements from individual cultures, n = 6. Statistical significance was assessed using the Student's t-test; *P < 0.05. (E) Cell immunoblot of COL1A1 and GAPDH from 14-day cultures. (F) Corresponding Immunoblot of COL1A1 released into the media over 3 days (days 11–14) with total protein for normalization. Quantitative PCR of (G) COL1A1 and (H) COL5A1 transcription relative to POLR2A. Points represent mean of individual donor cultures and shown as mean ± SEM; statistical significance was assessed using the Student's t-test; *P < 0.05, **P < 0.01. (I, J) Immunohistochemical localization of COL1A1, COL5A1 and FN1 in DN cultures and KC cultures. Green: protein of interest, Red: F-actin, Blue: DAPI. Scale bar: 50 μm.

Having established similar growth and survival of KC and DN cells in the 14-day LGSF system, we proceeded to test their ECM production capabilities. Picrosirius red was used to stain collagen and fast green to counterstain total protein (Figs. 2A, 2B). The KC cells appeared to have visibly reduced picrosirius red staining of the cell surface.

More sensitive methods of quantifying ECM production were then utilized. Hydroxyproline, being almost exclusively found in collagen was used to determine the total amount of collagenous material being produced by the cells. The hydroxyproline content was significantly lower (*P* = 0.045) in the KC cell layers compared to DN (*n* = 6) in LGSF, indicating a decrease in cell layer-associated total collagen in the patient keratocytes (Fig. 2C), whereas the media fractions showed similar hydroxyproline content (Fig. 2D).

Utilizing an antibody that is specific to the retained COL1A1 telopeptides, we detected three bands in Western blots of cell layer extracts, indicating active processing of procollagen as expected (Fig. 2E; Supplementary Figs. S3, S4). The KC samples showed consistent decrease in these cell-layer associated COL1A1 bands (DN = 2.5 ± 0.86, KC = 0.17 ± 0.17, *P* = 0.03). Unprocessed COL1A1 in the media, representing the “lost collagen” (Fig. 2F, Supplementary Fig. S5) showed a reduced trend (DN = 2.19 ± 0.23, KC = 1.56 ± 0.35, measured by densitometric scans of the bands,) but did not reach significance (*P* = 0.18). Transcriptional levels of *COL1A1* and *COL5A1* were also reduced in KC (Fig. 2H) cells relative to DN (Fig. 2G), (*COL1A1 =* 0.03 ± 0.01, *COL5A1 =* 0.16 ± 0.05). KC keratocytes also show reduced ECM staining for COL1A1, COL5A1 and fibronectin (FN1; Figs. 2I, 2J; Supplementary Fig. S6).

### Collagen Processing Proteins Are Downregulated, and Catabolic Pathways Upregulated in KC Keratocytes

**Figure 3:**
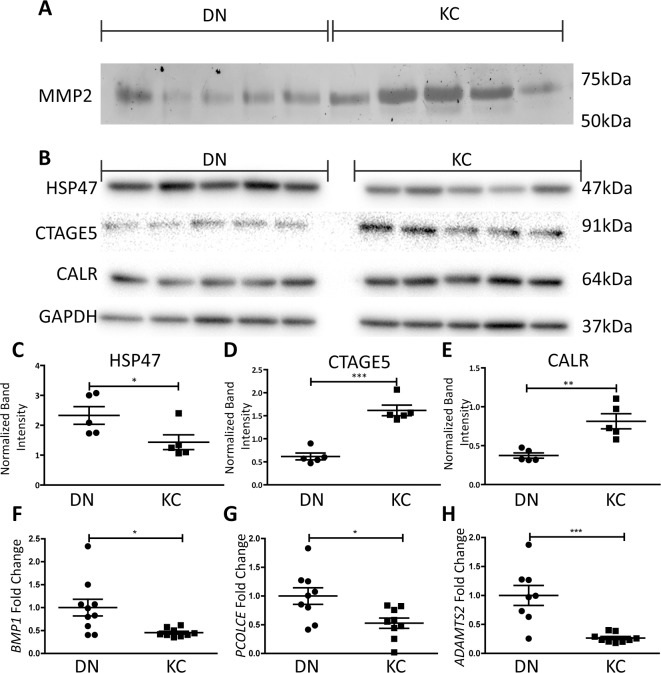
Increased MMP2 and decrease in collagen maturation related proteins in KC keratocytes. (A) Gelatin zymography of day 14 media (days 11–14) with bands corresponding to 65 kDa latent MMP2, and 60 kDa active MMP2 in 6 DN and 6 KC patients. (B) Western blots of HSP47, CTAGE5 and CALR and GAPDH as internal control. (C–E) Quantification of HSP47, CTAGE5, CALR, points represent mean of individual donor cultures and range is standard error of mean. Values are shown as mean ± SEM; statistical significance was assessed using the Student's t-test; *P < 0.05, **P < 0.01, ***P < 0.005. (F–H) Quantitative PCR of BMP1, PCOLCE, ADAMTS2, using 2-ΔΔcT relative to POLR2A, n ≥ 9 biological repeats.

We sought to determine if decreased ECM in KC keratocytes was due to increased matrix metalloproteinases and ECM degradation. Gelatin zymography showed increased MMP2 in day 14 KC cell culture media (Fig. 3A; Supplementary Fig. S7), 11,830 ± 1799 vs. 21,490 ± 3756 arbitrary units, in DN and KC, respectively. We next investigated whether collagen maturation and processing pathways were at fault, as this could contribute to misfolded protein, feedback transcriptional decrease and decreased collagen output. HSP47, an obligate collagen chaperone was significantly reduced in KC cells,^29^ 2.33 ± 0.29 vs. 1.43 ± 0.25 (Figs. 3B, 3C; Supplementary Fig. S8) Western blots show that CTAGE5/TANGO1,^30^ required for loading large (>70 nm) cargo into COPII vesicles for export was increased, 0.62 ± 0.07 vs. 1.62 ± 0.12 in KC cell culture extracts (Fig. 3D). The endoplasmic reticulum protein CALR,^31^ which binds calcium and unfolded proteins was also found to be increased in KC keratocytes, 0.37 ± 0.03 vs. 0.81 ± 0.01 (Fig. 3E; Supplementary Fig. S8). Transcripts for *BMP1*,^32^ the procollagen C-terminal protease required in collagen maturation and assembly was decreased 0.45 ± 0.03 (Fig. 3F). *PCOLCE*,^33^ a protein which enhances the activity of BMP1 was decreased 0.53 ± 0.09 (Fig. 3G). *ADAMTS2*,^34^ the procollagen N-terminal protease was also decreased 0.27 ± 0.03 (Fig. 3H). These results suggest that the collagen maturation was adversely affected in KC keratocytes in culture. Moreover, increased MMP2 could be contributing to increased degradation of any properly folded and secreted collagen.

### Global MS Reveals Altered Stress Response

**Figure 4:**
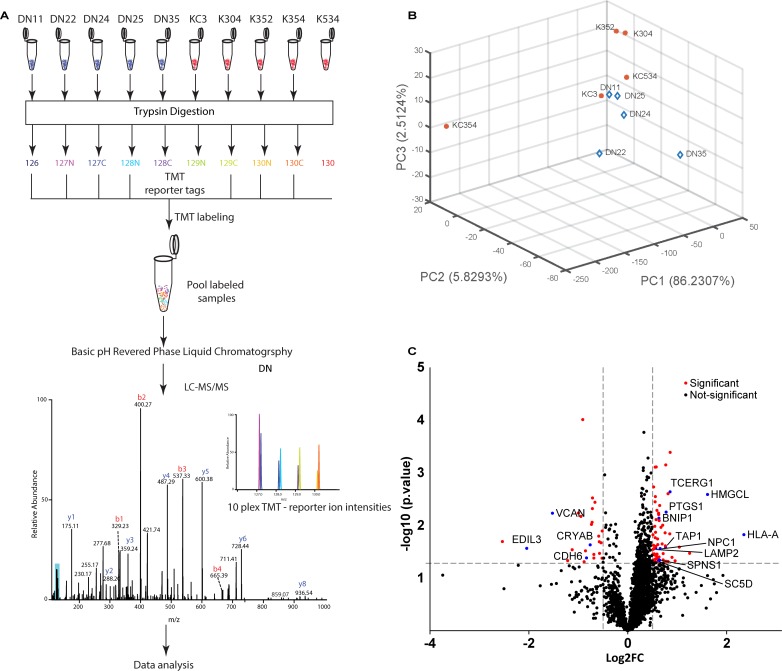
Increased stress related proteins in the KC keratocyte proteome. (A) Methodology of TMT labeled tandem mass spectrometry. (B) Principal component analysis of 5DN and 5KC samples showing KC354 with large deviation from the other samples. (C) Volcano plot of significantly changed proteins (red) 89 proteins were significantly altered those discussed in the text are highlighted in blue.

For an understanding of global proteomic changes in KC keratocytes we undertook multiplexed mass spectrometry on 5 DN and 5KC keratocyte cultures maintained in the 14-day LGSF system (Fig. 4A). A principal component analysis (PCA), based on the proteomic profile identified one KC sample as significantly distant from the other KC samples and removed from the remaining analyses (Fig. 4B; Supplementary Fig. S9). A total of 2796 proteins were detected. Among these, several stromal ECM proteins, COL1A1, COL1A2, COL3A1, COL5A1, COL5A2 and proteoglycans LUM, DCN, and FMOD (Table, a complete list of detected proteins may be accessed at https://www.ebi.ac.uk/pride/, in the public domain), were decreased in the KC cell proteome, as already revealed by our biochemical analyses for some of these. A set of 89 proteins was considered significantly and consistently different between DN and KC samples (Fig. 4C; Supplementary Fig. S10). These include upregulations that suggest increased restriction on translation (transcription elongation regulator 1, TCERG1), glucose-alternative means of energy procurement—ketogenesis (HMGCL), prostanoid biosynthesis (PTGS1), cholesterol (SC5D) biosynthesis and transport (NPC1), peptide degradation and transport (LAMP2, TAP1, HLA-A), autophagy and cytoprotection (SPNS, BNIP1). Decreases, on the other hand, include ECM and cell adhesion proteins (VCAN, EDIL3, CDH6), and the molecular chaperone CRYAB. Overall, this scenario of translational inhibition, altered metabolism and increased protein degradation, conforms to an integrated stress response (ISR) pattern in KC cells.

**Table:**
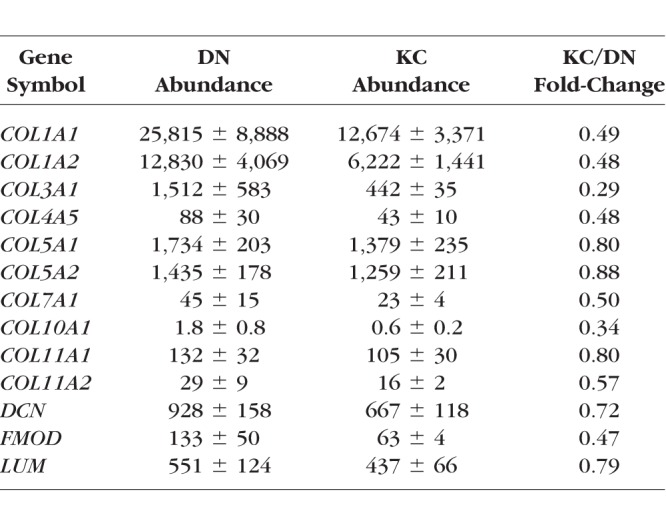
Altered ECM Proteins Selected From MS Analysis

### Persistent Activation of the Integrated Stress Response (ISR) in KC

**Figure 5:**
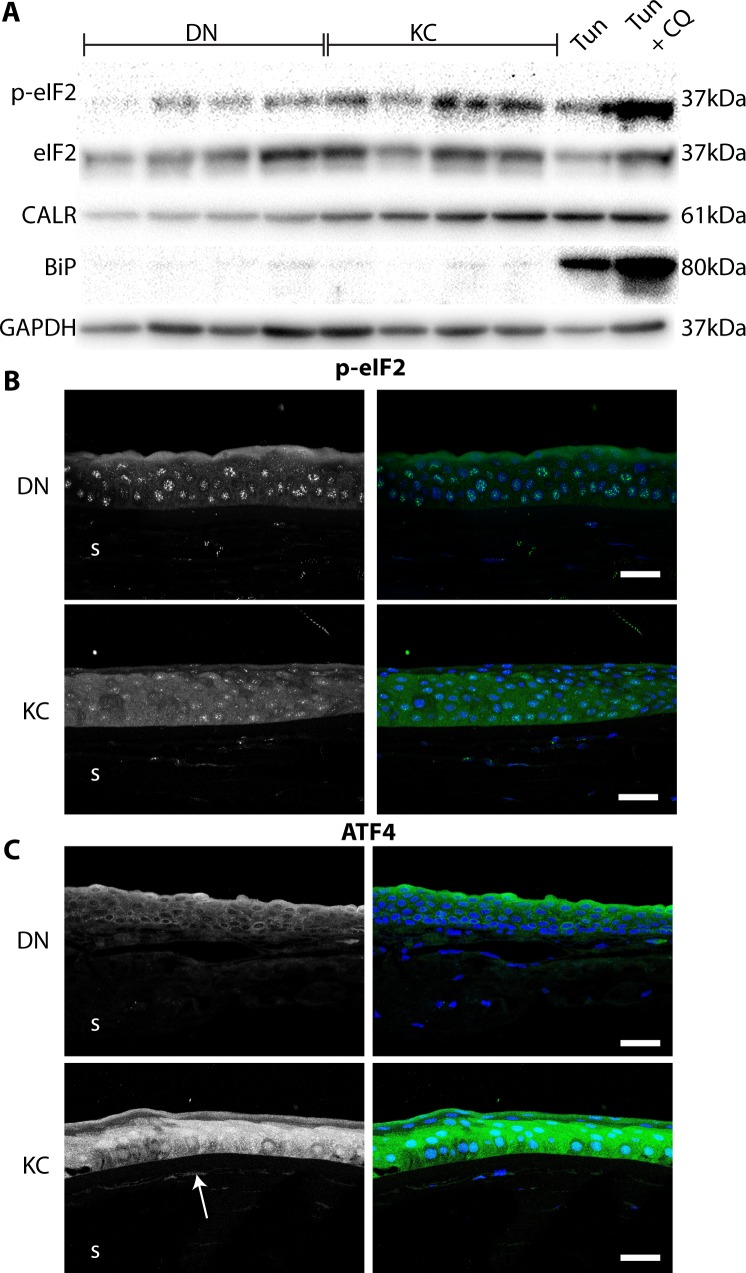
Integrated stress response pathway peIF2α and ATF4 increased in KC. (A) Immunoblots of eIF2α, BIP, CALR, and GAPDH in fibroblasts from 4DN and 4 KC samples, with positive controls of donor fibroblasts treated for 24 hours with 1 μg/mL Tunicamycin (Tun), and Tunicamycin + 40 μg/mL Chloroquine (Tun+Cq). (B) Representative immunohistochemistry of phosphorylated eIF2α in DN and KC corneas. (C) Representative immunohistochemistry of ATF4 in DN and KC cornea control, White arrow denotes ATF4 in subepithelial stroma. Scale bar: 50 μm.

ISR is initiated through phosphorylation at Ser 51 and activation of the translation elongation initiation factor subunit α (eIF2α) which down regulates protein synthesis to conserve energy^20^, but upregulates activating transcription factor (ATF4) mediating transcriptional programs underlying metabolism, autophagy and cell survival.^19,35,36^ We tested if KC cells, even without a serum-starvation challenge, have elevated p-eIF2α. Thus, extracts of KC fibroblasts show elevated p-eIF2α (0.35 ± 0.03 KC vs. 0.18 ± 0.03 DN), accompanied with elevated CALR (0.35 ± 0.02 vs. 0.69 ± 0.1; *P* = 0.02) as seen before for KC keratocytes. In contrast, DN cells show elevated p-eIF2α only after tunicamycin mediated blocking of protein N-glycosylation and export (Fig. 5A, last two lanes; Supplementary Fig. S11).

We tested if p-eIF2α staining was increased in KC corneal sections. We identified diffuse punctate staining of p-eIF2α in DN samples, whereas the KC samples had much more robust staining in both the epithelium and the stromal keratocytes (Fig. 5B; Supplementary Fig. S12). Consistent with the cell culture data, the downstream effector of persistent p-eIF2α, ATF4 staining was increased, with increased nuclear localization in KC corneal sections. This pattern was observed in the epithelial and the sub-epithelial stromal layers (Fig. 5C).

## Discussion

An important step toward understanding pathogenesis of a disease is the establishment of cell culture models. Of course, this does not obviate the importance of animal models. For example, the *Col5a1*^+/−^, haplo-insufficient for collagen type V,^37^ and the Lum^−/−^ mice have thin corneas and reduced corneal collagen.^38,39^ The *Kera*^−/−^ mice have thin corneas as well.^40^ Collectively, these and other genetically altered mice are important tools for exploring certain aspects of keratoconus. However, the advantage of a cell culture model is that it allows readouts of a disease that can be assessed with relative ease and utilized to develop screens for candidate genes and therapeutic factors. Here, building on our previous corneal stromal keratocyte culture studies,^22,23^ we developed an approach to isolate stromal cells from keratoconus corneas, expand these as fibroblasts and push them to a keratocyte-like phenotype in a serum-free low glucose medium. Within a 14-day span of culture in this restricted medium, KC cells were no different from DN keratocytes in cell survival. However, they clearly lacked the ability to produce or sustain an ECM comparable to that seen in DN cells. We used several independent assays to demonstrate that the fibril-forming collagen type I was reduced. An important and practical outcome of these experiments is the establishment of an approach for assessing collagen as a functional readout of keratoconus cells.

**Figure 6:**
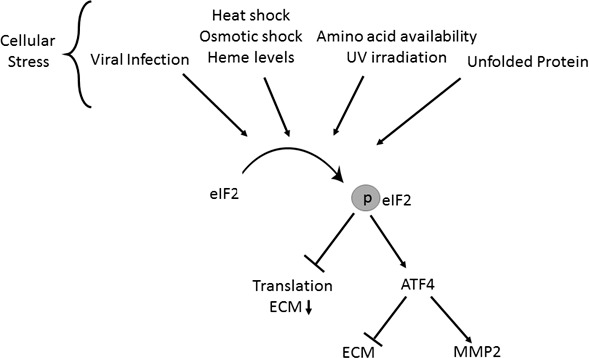
Schematic diagram of the stress response induced in KC cells.

Our 14-day LGSF culture system showed overall lower levels of KC keratocyte-associated collagens (Sirius red and hydroxyproline assays) and more specifically lower collagen type I levels. Both fibronectin and collagen type V matrices were qualitatively decreased and altered as well. These ECM decreases were accompanied by decreases in protein and transcripts for HSP47, *BMP1*, *PCOLCE,* and *ADAMTS*2, indicating a feedback downregulation in collagen chaperone, and processing enzymes to meet the reduced “collagen demand.”^29,32,33,41–43^ A mass spectrometric global proteome profiling of the cell cultures reaffirmed decreases in ECM proteins. But of the most significant and consistent changes seen in KC compared to the 5 DN cell proteomes suggest an exaggerated effort in KC cells to inhibit protein translation and use ketogenesis for procuring energy from fatty acids in the glucose restricted environment. Therefore, we questioned whether the ECM decreases in KC cells were a part of an eIF2α/ATF4 integrated stress response pathway, whereby the cell decreases non-essential mRNA translation via phosphorylation and activation of the translation initiation factor eIF2α (Fig. 6). The transcription factor, ATF4, in turn selectively up regulates MMPs and pro-survival genes.^36^ Indeed, we found increases in p-eIF2α and ATF4 in KC cell extracts and in tissues sections of KC corneas. The activation of the ISR is also known to up regulate transport proteins, important in the clearance of unfolded proteins. This may explain the increase of transport proteins (CALR and CTAGE5) we observe coupled with the altered dynamics of lysosomal proteins.

Further studies are needed to understand why KC stromal cells are intrinsically poised for ISR. The cornea is a unique tissue in that it is conceivably exposed to multiple environmental stresses, ranging from UV exposure, heat shock to microbial and viral infections. Many of these stresses have been reported in keratoconus.^15,44,45^ Iron imbalance and accumulation of amyloid products has also been known to be affected in keratoconic corneas for more than 50 years.^46^ More recently altered cytokine balances in the tear film and increased presence of iron chaperones have been reported in keratoconus as well.^17,47,48^ Given that it is avascular, the supply of cellular metabolites is tightly regulated, and conditions that may be tolerated elsewhere become pathogenic in the cornea. Our study underscores the idea that keratoconus is precipitated by multi-gene and environmental interactions, bringing integrated stress response related genes into the arena of possible genetic candidates.

## Supplementary Material

Supplement 1
