# Supplementary material for: Integrated Stress Response and Decreased ECM in Cultured Stromal Cells From Keratoconus Corneas
Source: Invest Ophthalmol Vis Sci. 2018 Jun;59(7):2977–86. doi: 10.1167/iovs.18-24367 (PMC5995483; doi:10.1167/iovs.18-24367)
Supplement: Supplement 1 [file iovs-59-06-59_s01.pdf]

Supplemental table 1. Sample Information

Sample	Sex	Age	Ancestry	A/K score	Use
DN 2	M	56	Caucasian		WB
DN 9	Un	Un	Un		WB
DN 10	Un	Un	Un		WB
DN 11	Un	26	Un		PCR, WB, MS
DN 15	Un	68	Un		PCR, Zy, Hy, SR
DN 17	F	89	Caucasian		WB
DN 21	M	48	Caucasian		WB
DN 22	M	21	Un		PCR, WB, MS
DN 24	M	53	Caucasian		PCR, WB, MS
DN 25	F	59	Un		PCR, WB, Hy, SR, MS
DN 26	F	54	Caucasian		PCR, Hy, SR
DN 31	M	71	Caucasian		PCR, WB
DN 35	M	42	Caucasian		MS
DN 50	Un	Un	Un		PCR, IHC, Hy, SR
DN 88	Un	Un	Un		PCR, Zy, Hy, SR
DN 89	Un	Un	Un		PCR, Zy, Hy, SR
K 0413	F	44	AA	3	PCR, Zy, Hy, SR
K 0913	F	22	AA	4	PCR, WB
K 2198	M	23	Hispanic	4	PCR, Zy, Hy, SR
K 2807	M	76	Caucasian	4	PCR, Zy, Hy, SR
K 2912 (K3)	M	50	Caucasian	Un	PCR, WB, Zy, Hy, SR, MS
K 304	F	23	AA	4	PCR, WB, Zy, Hy, SR, MS
K 352	F	47	Caucasian	3	PCR, WB, MS
K 354	F	22	AA	3	MS
K 534	F	22	AA	4	PCR, WB, Zy, Hy, MS
K 118 (KC7)	M	22	Middle Eastern	4	WB
K 1209	M	44	AA	3	WB
K 2112	M	53	Caucasian	Un	WB
K 2805	M	76	Caucasian	4	WB

A/K score = Amsler-Krumeich score, UN = Unknown
PCR = quantitative PCR, WB = Western Blot, IHC = Immunocytochemistry, Zy = Zymography, SR = Sirius Red, MS = Mass spectrometry, HY = Hydroxyproline

Supplemental table 2 - Antibodies used

Target	Catalogue number	Provider	Raised in	Dilution
COL1A1 (WB)	AF6220	R&D systems	Sheep	1:2000
COL5A1	nbp1-19633	Novus	Rabbit	1:500
FN1	ab2413	Abcam	Rabbit	1:2000
GAPDH	G8795	Sigma-Aldrich	Mouse	1:5000
ALDH3A1	ab76976	Abcam	Rabbit	1:1000
CALR	Ab2907	Abcam	Rabbit	1:1000
CTAGE5	Ab154369	Abcam	Rabbit	1:1000
HSP47	ab109117	Abcam	Rabbit	1:1000
Phospho ser51 eif2	3398	Cell Signalling	Rabbit	1:500
eIF2	5324	Cell Signalling	Rabbit	1:1000
BiP	3177	Cell Signalling	Rabbit	1:1000
HRP anti sheep	HAF016	R&D systems	Donkey	1:5000
HRP anti rabbit	7074	Cell Signalling	Donkey	1:5000
HRP anti goat	SC-2020	Santa-Cruz	Donkey	1:5000
HRP anti mouse	7076	Cell Signalling	Donkey	1:5000
Alexa 488 anti rabbit	A21206	Life Technologies	Donkey	1:500

Supplemental table 3 - qPCR primers used

Gene	Forward	Reverse	Size (bp)	melt temp °C
ADAMTS2	ACGCCAAGCACTGCAATG	AACGACCAGGGCAGAGCTC	97	83.7
BMP1	ATAACTCGGTCCAGCGAAAGG	AGTTGTTGTCGCCAAACTGG	120	82.3
COL1A1	GTCCTAAAGGTGCCAATGGT	ACCAGGTTCACCGCTGTTAC	128	87.7
COL5A1	TCAGGCAAGTTGTGAAAATCT	CCATACCCGCTGGAAAGC	150	83.4
HSP47	TGCTAGTCAACGCCATGTTC	ATCATGACACCCACGGTATAGG	112	81.3
KERA	TGG GCT GGG ACA TAT TAC AGA	TCA ACT CAC AAA GGT TCC CC	103	77.7
PCOLCE	CAGACCCCCAACTACACCAG	GTTGGGGAACCCCTCACT	90	82.3
THY1	GGGAGACCTGCAAGACTGTT	CGGAAGACCCCAGTCCA	94	82
RNAP2A	CATCATCCGAGACAATGGTG	AACAATGTCCCCATCACACA	115	82

Supplemental figure 1 - Cell morphology day 14

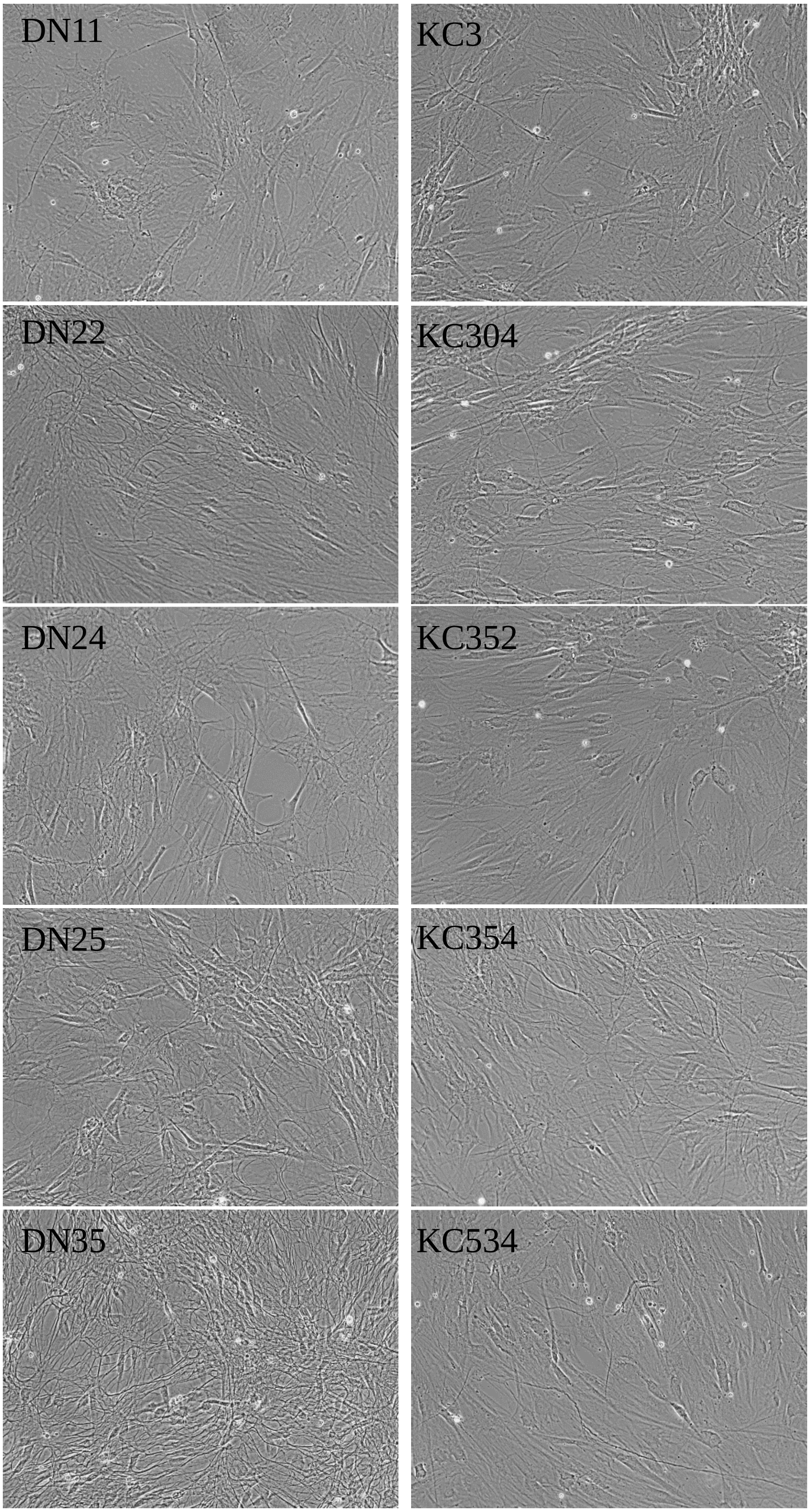

Supplemental Figure 2 - Western blots for figure 1

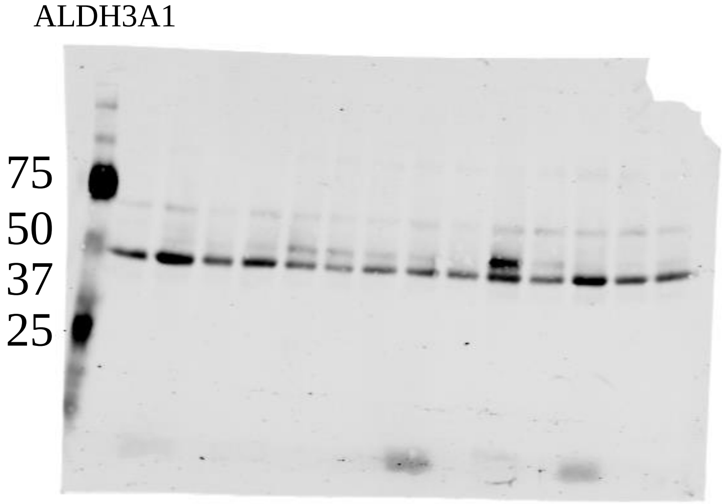

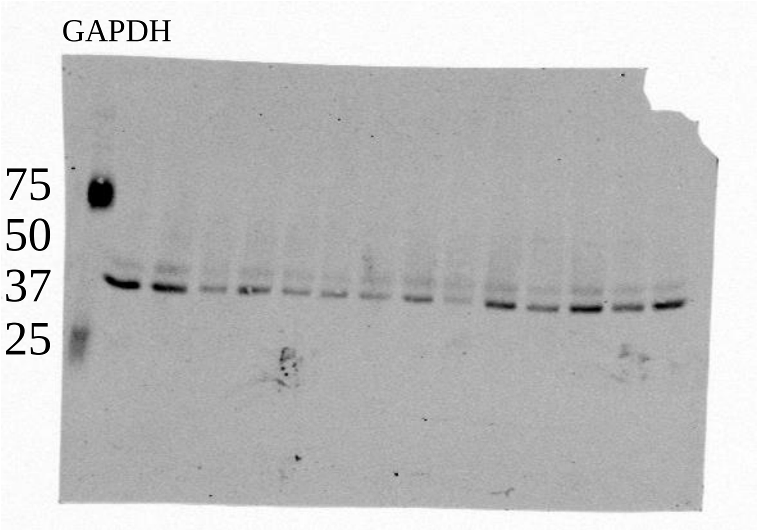

1. Ladder
2. DN 2
3. DN 9
4. DN 10
5. DN 11
6. DN 17
7. DN 21
8. DN 22
9. KC 118
10. KC 1209
11. KC 7 12. KC 2805
13. KC 304
14. KC 2112
15. KC 2912

Supplemental figure 3 - COL1A1 antibody validation for figure 2

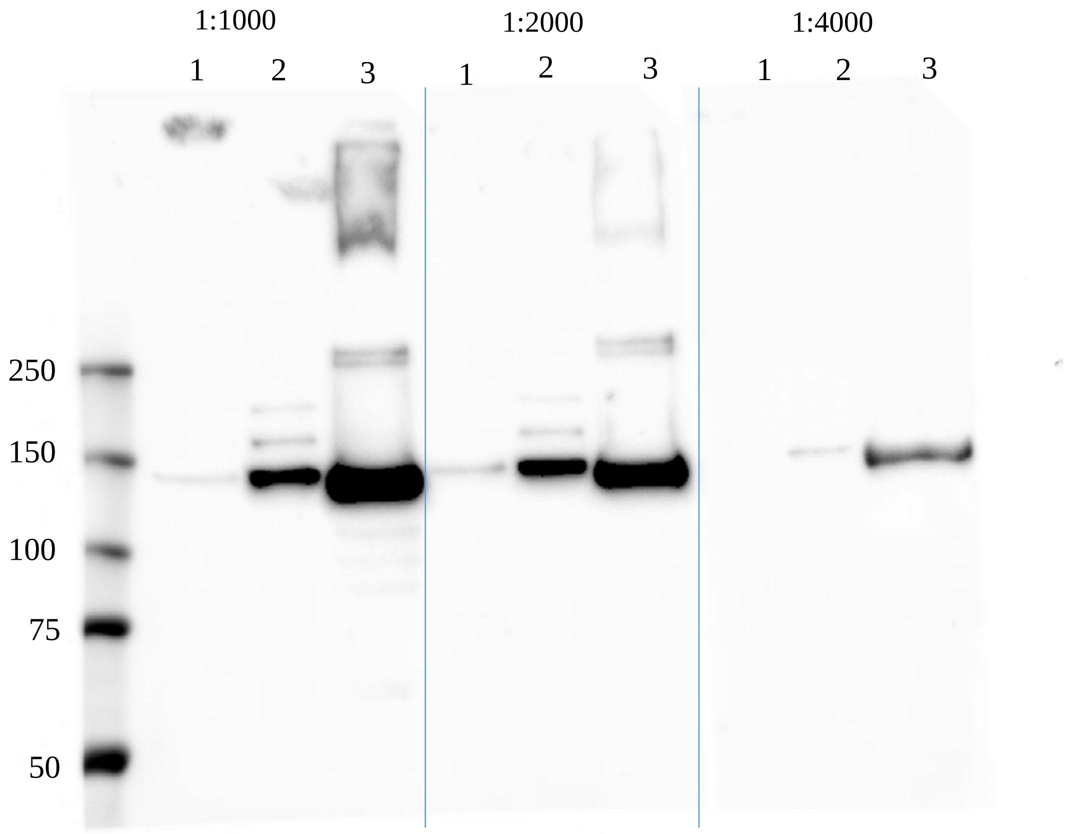

1. Cornea extract
2. D14 cell layer
3. Human placental collagen

Immunogen

Chinese hamster ovary cell line CHO-derived recombinant human Collagen I a1Gln23-Lys277, Gly1094-Leu1464Accession # P02452

7% gel
Boiled, reducing
5% NF milk in PBS
O/N 4 deg. PBST (0.1%) 5% NF milk
R&D - AF6220
1:5000 2nd ab 1hr RT 5% NF milk

Supplemental figure 4 - Cell layer immunoblots figure 2

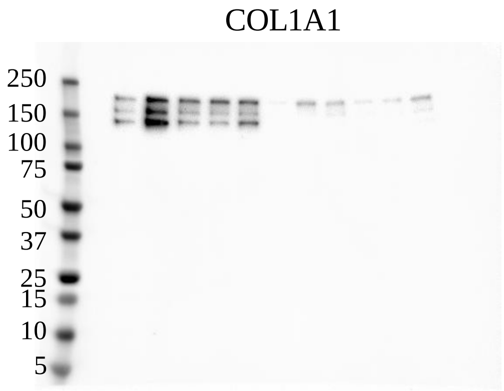

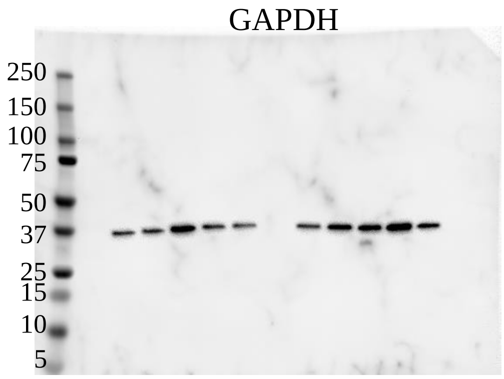

1.	Ladder	8. Blank
2.	Blank	9. KC3
3.	DN11	10. KC0913
4.	DN22	11. KC304
5.	DN24	12. KC352
6.	DN25	13. KC534
7.	DN31	14. Blank

Expected sizes:
COL1A1 – 130-180kDa
HSP47 – 47kDa
GAPDH – 37 kDa

Supplemental figure 5 - Media immunoblots, figure 2

COL1A1

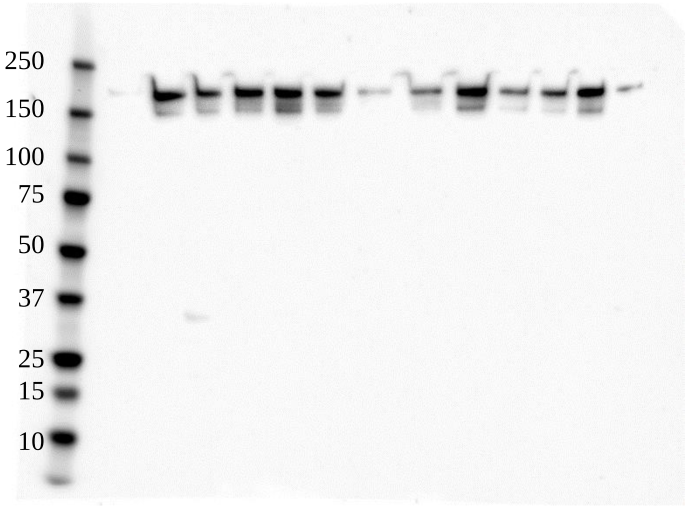

Stain free

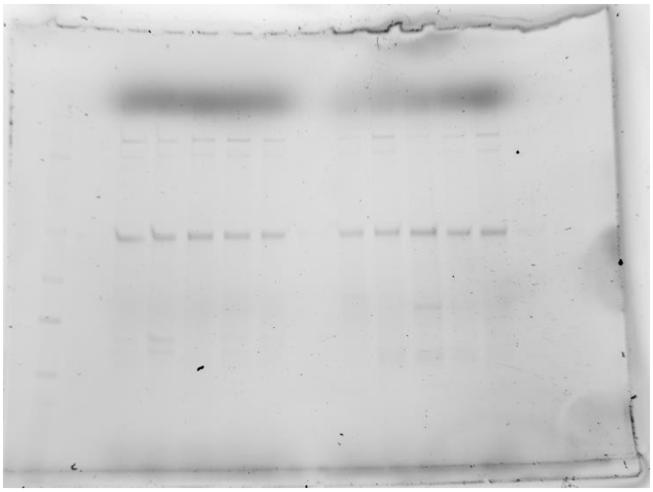

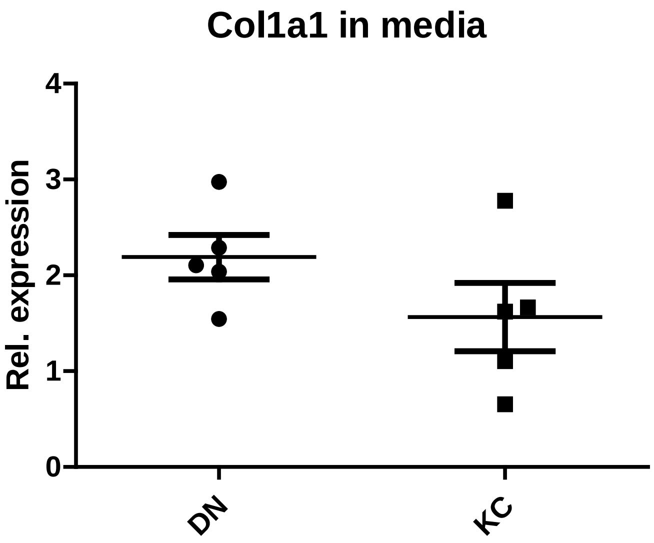

1. Ladder 8. Blank
2. Blank 9. KC3
3. DN11 10. KC0913
4. DN22 11. KC304
5. DN24 12. KC352
6. DN25 13. KC534
7. DN31 14. Blank

Expected sizes:
COL1A1 – 130-180kDa

Supplemental figure 6 – Immunohistochemistry for figure 6 & 8

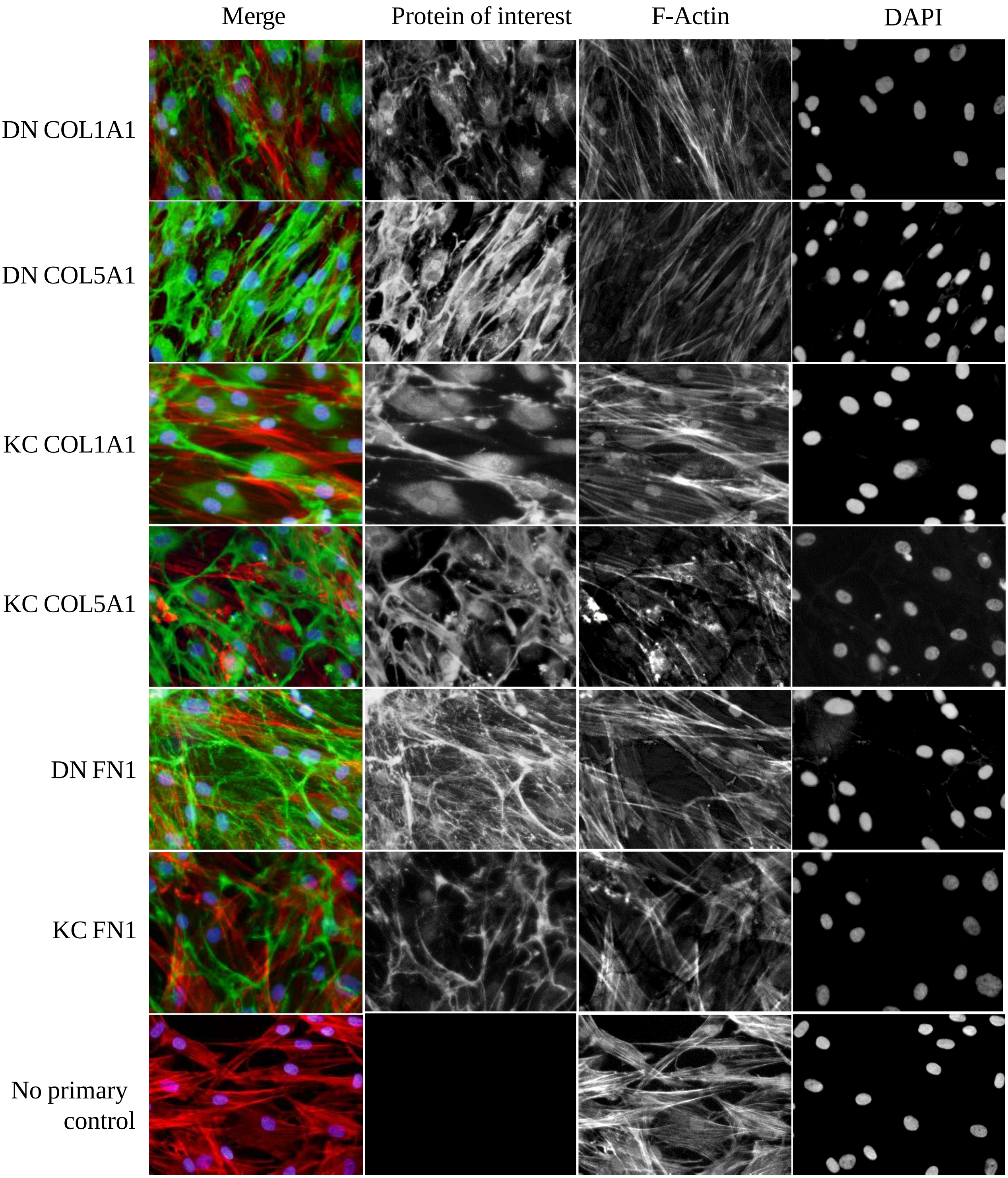

Supplemental figure 7 –Gelatin Zymography for figure 3

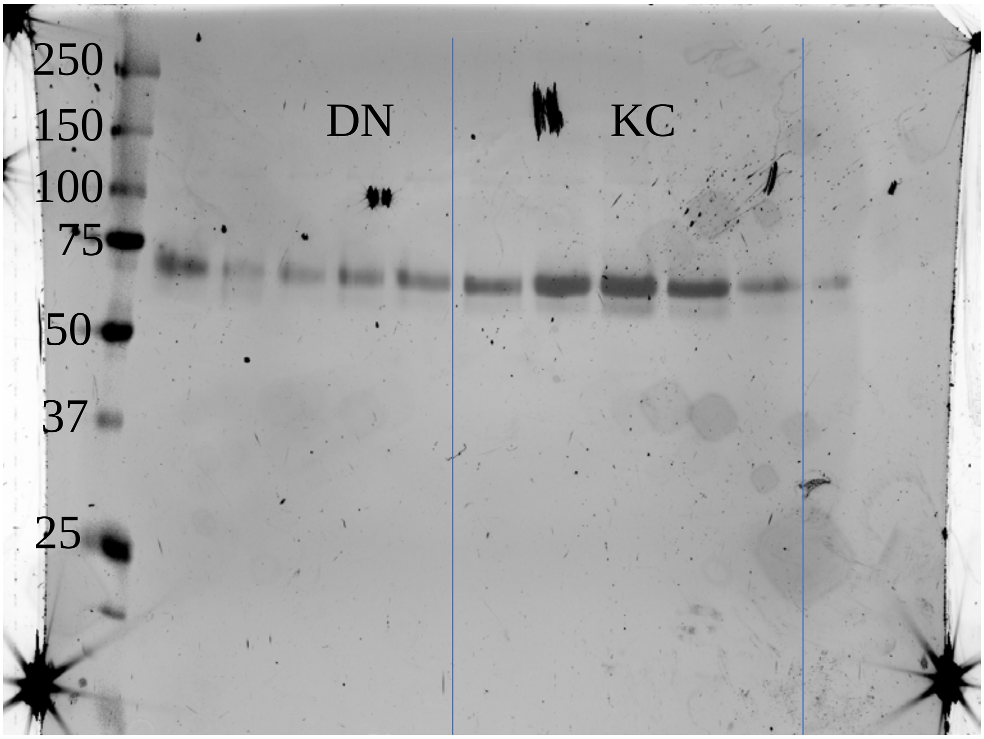

Table Analyzed	Data 1
Column A	DN
vs	vs
Column B	KC
Unpaired t test	
P value	0.0490
P value summary	*
Are means signif. different? (P < 0.05)	Yes
One- or two-tailed P value?	Two-tailed
t, df	t=2.319 df=8
How big is the difference?	
Mean ± SEM of column A	11830 ± 1799 N=5
Mean ± SEM of column B	21490 ± 3756 N=5
Difference between means	-9659 ± 4165
95% confidence interval	-19260 to -55.18
R squared	0.4020

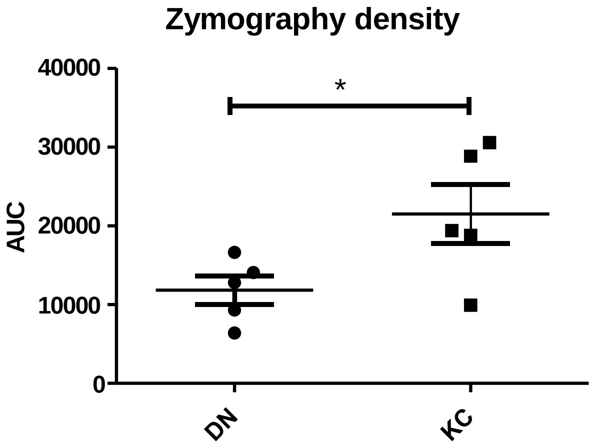

Supplemental figure 8 - Cell layer immunoblots figure 3

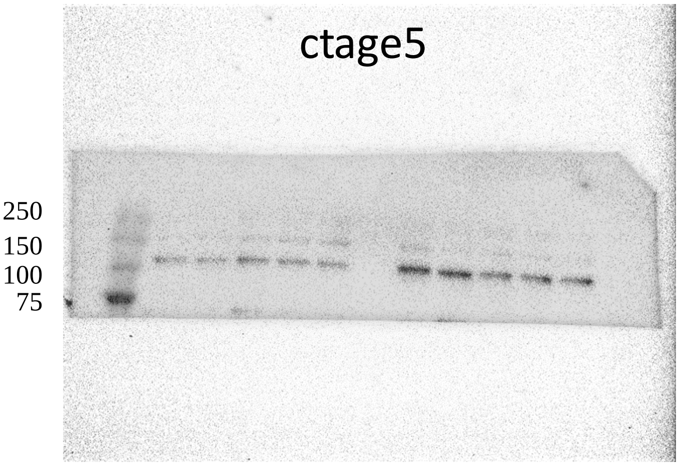

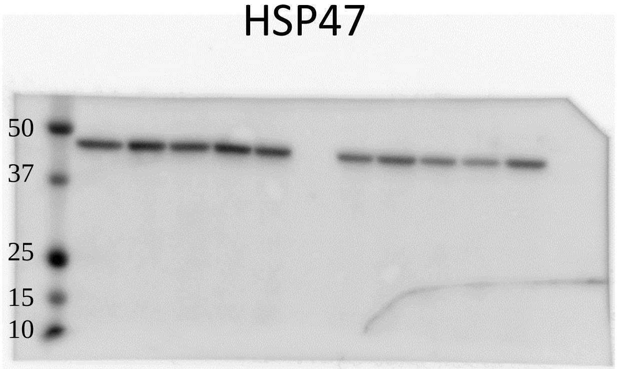

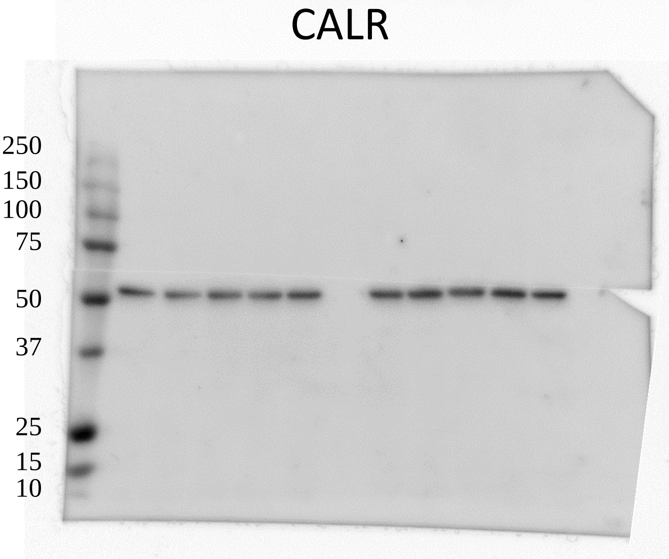

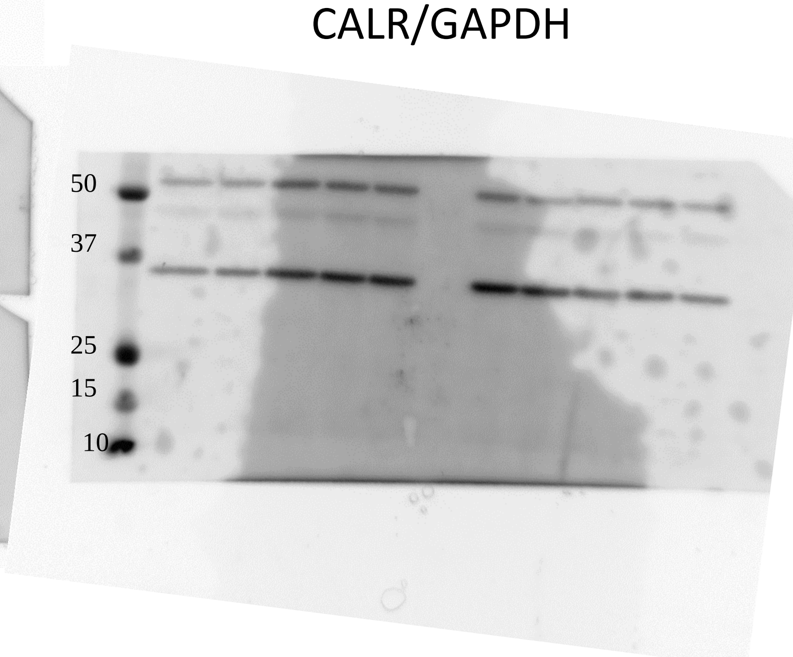

1. Ladder
2. DN11
3. DN22
4. DN24
5. DN25
6. DN31
7. Blank
8. K3
9. KC0913
10. KC304
11. KC352
12. KC534
13. Blank

Expected sizes:
CTAGE5 – 91kDa
HSP47 – 47kDa
CALR – 64kda
GAPDH – 37 kDa

Supplemental Figure 9 – initial MS analysis and validation

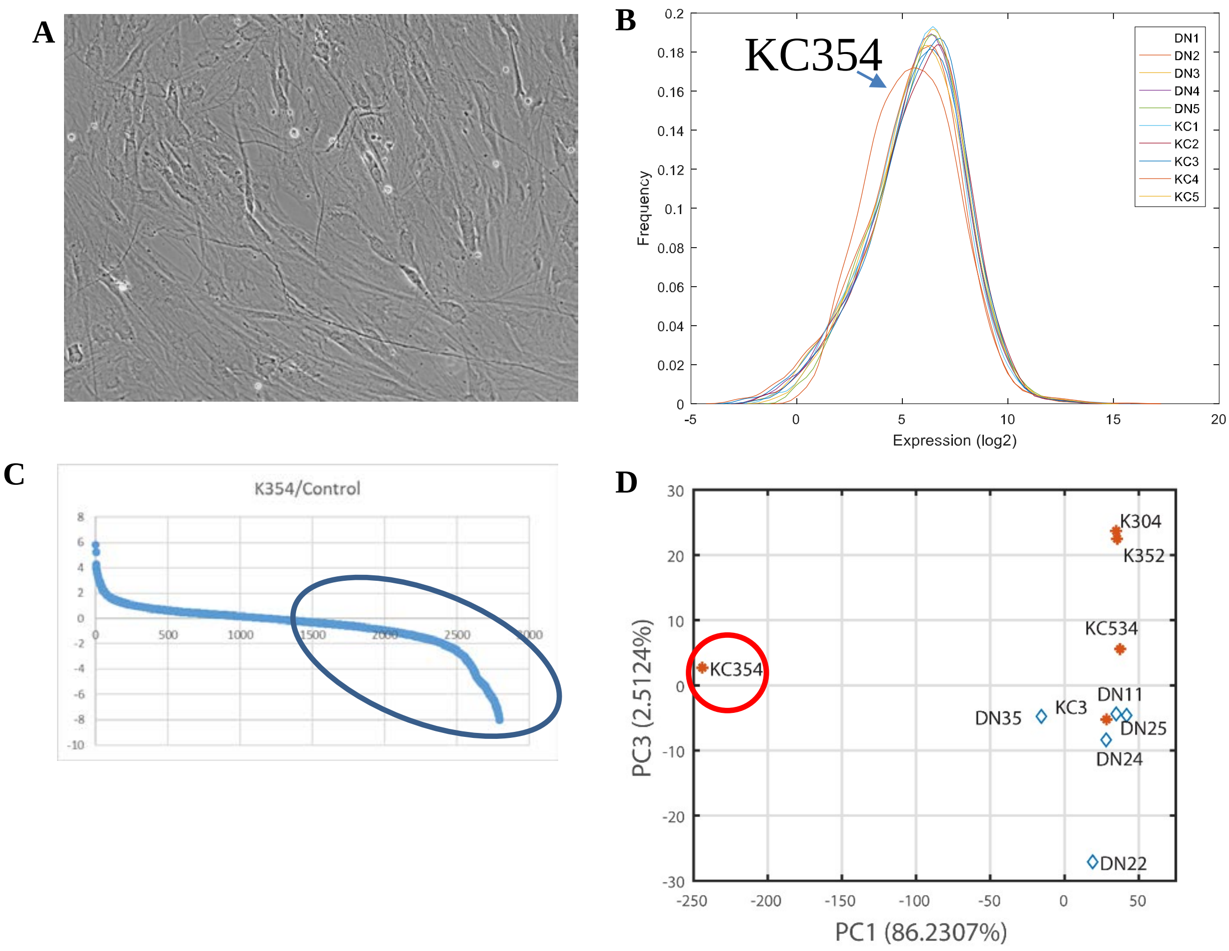

KC354 Appeared normal under brightfield microscopy (**A**), but protein frequency plots were skewed towards proteins of low expression (**B**) with a flattened distribution not seen in the other samples.

Plotting the abundance profile also demonstrated a non-normal expression distribution (**C**). PCA analysis also showed that KC354 was did not cluster well with the other samples (-200). On these basis we decided to exclude KC354 from further analysis.

Supplemental Figure 10 –Continued MS analysis

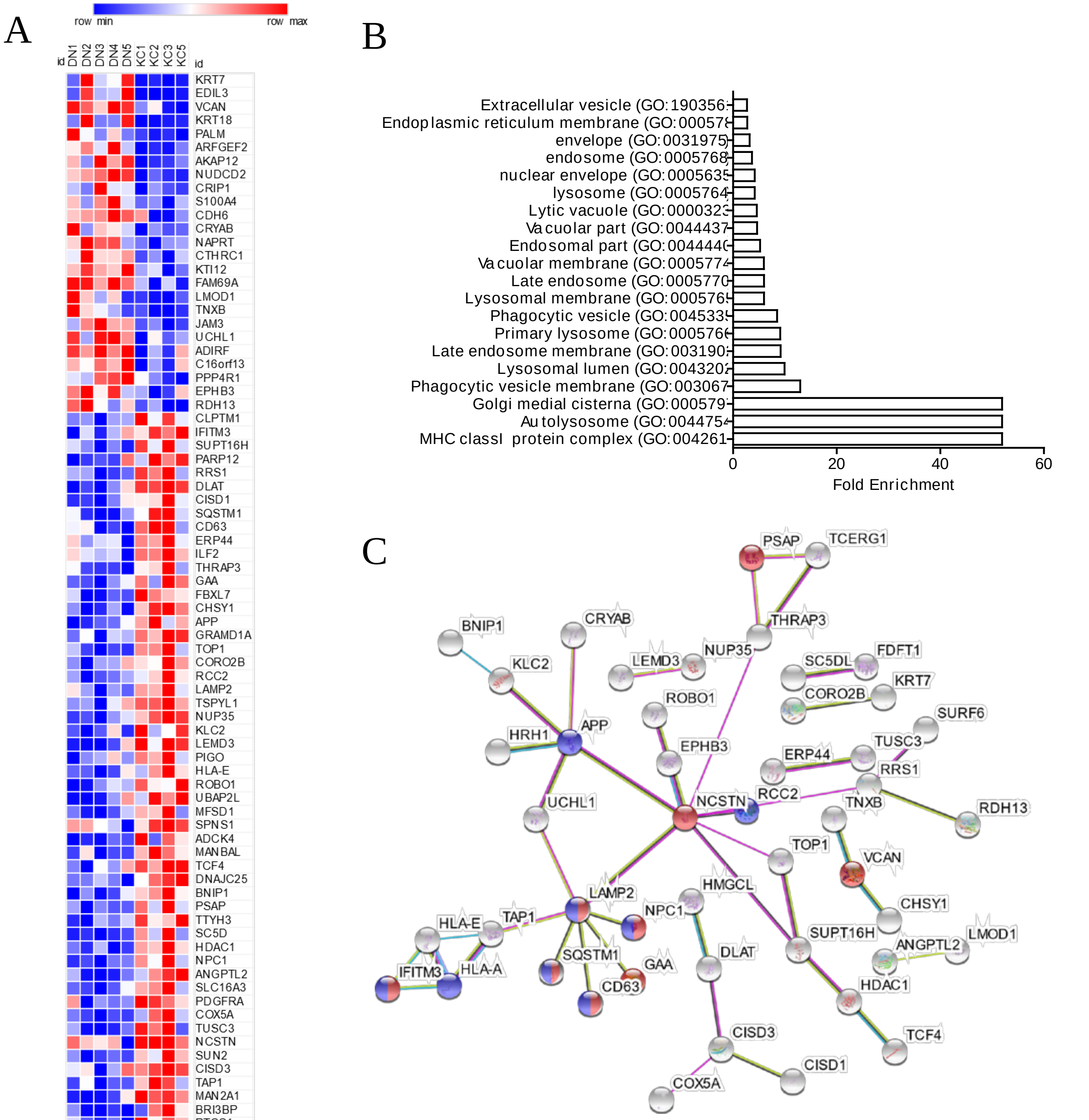

A. Heat map of most changed (89) proteins from cell culture proteomics.\
B. GO overrepresentation analysis (PANTHER) showing enriched protein classes.

C. STRING-DB analysis of interacting proteins. Red – lysosomal proteins, Blue, endosomal proteins.

Supplemental figure 11 - Cell layer immunoblots figure 4

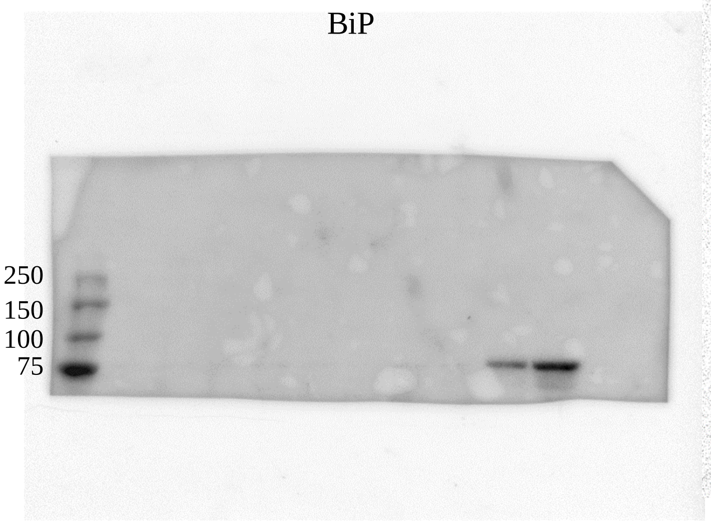

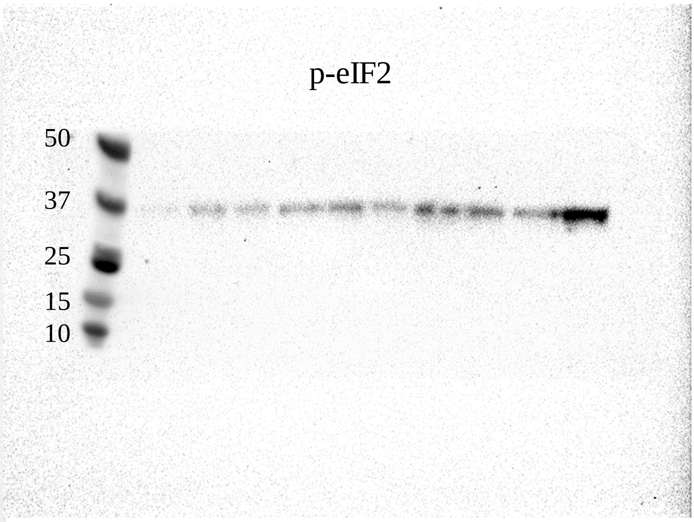

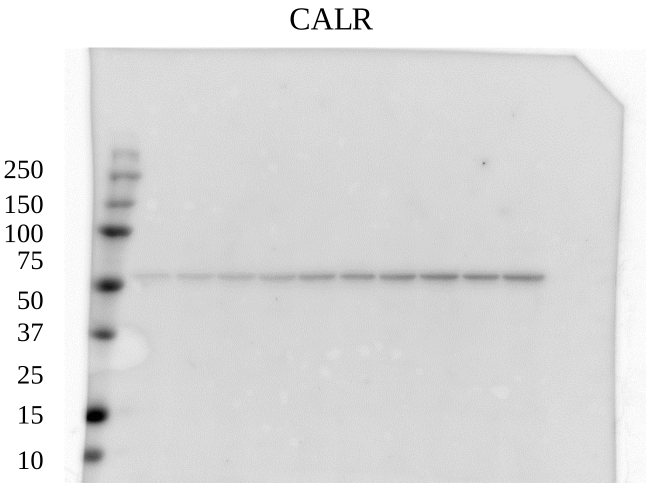

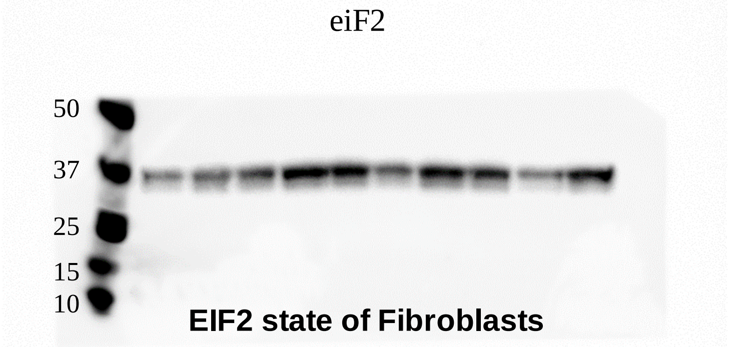

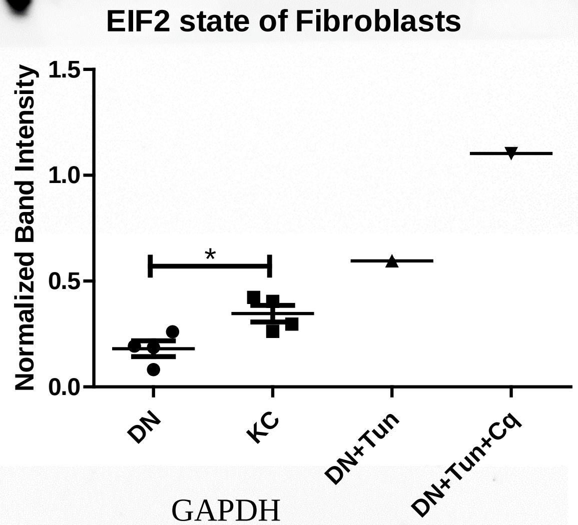

Expected sizes:
eIF2/p-EIF2 – 37kDa
CALR – 61kDa
BiP - 80kDa
GAPDH – 37 kDa

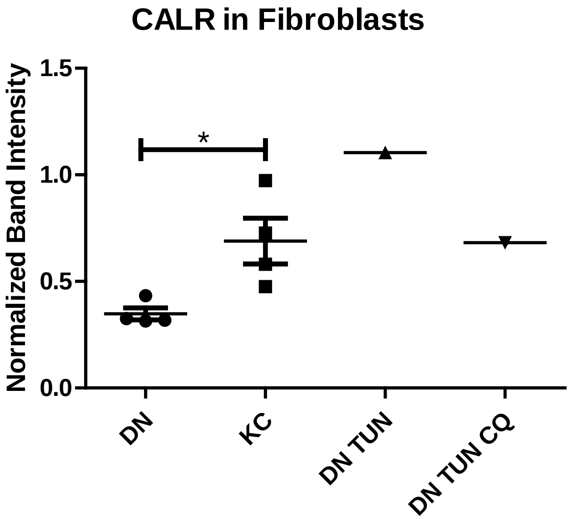

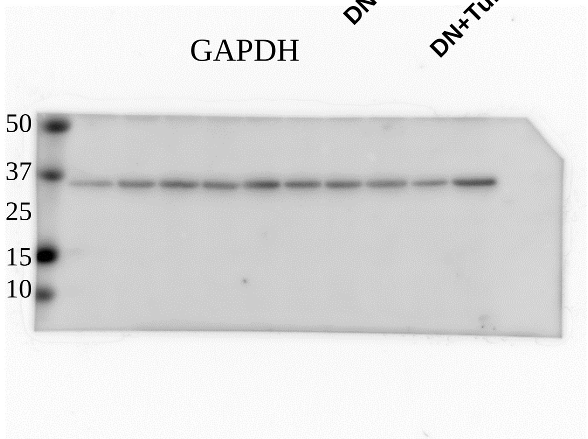

Supplemental Figure 12, immunohistochemical staining for integrated stress response proteins in DN and KC corneas

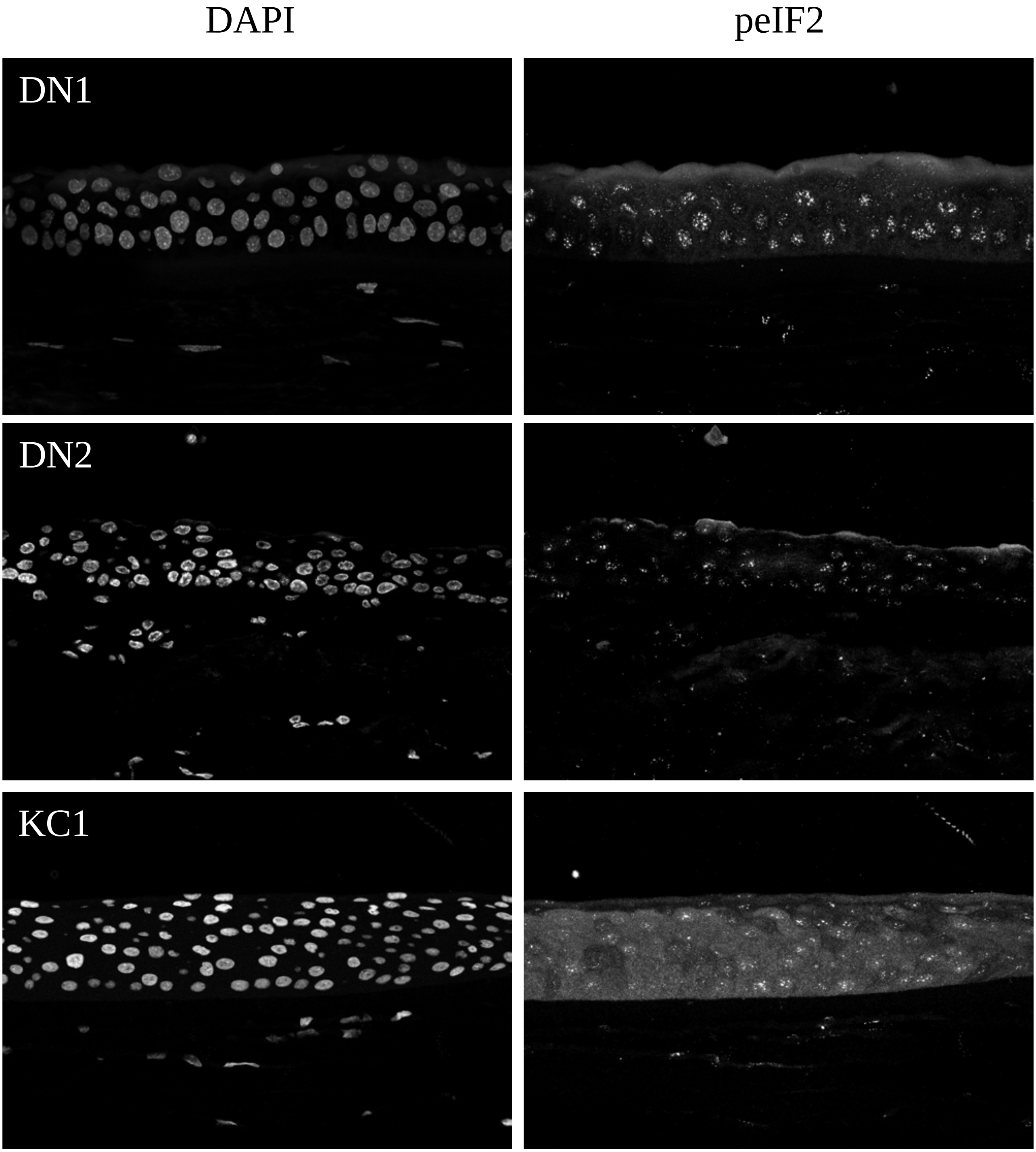

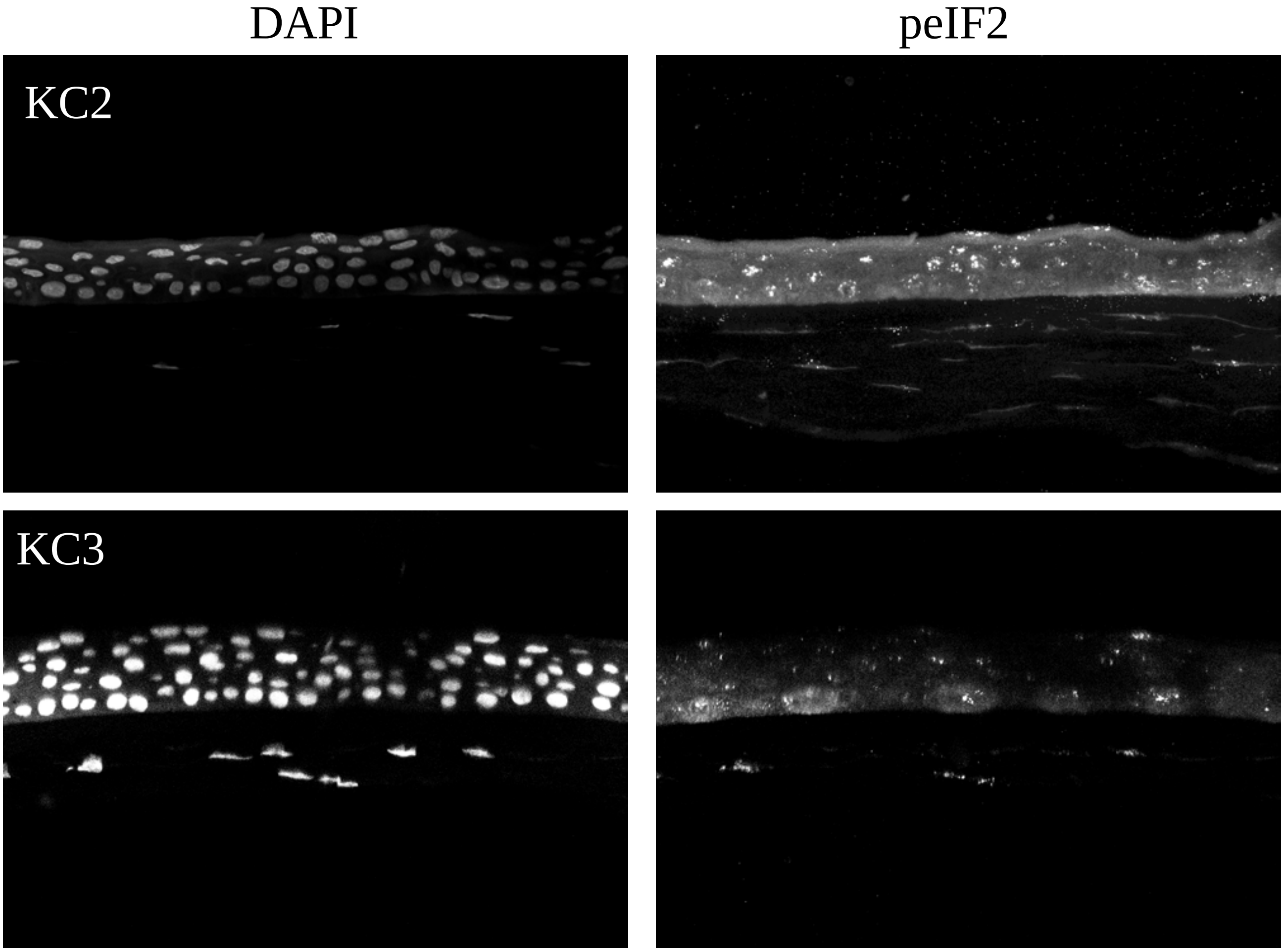

P-eIF2, Abcam ab32157. 1:200 in TBS 5% BSA, overnight 4°C.
Tris-EDTA pH 8.0 antigen retrieval, 20mins 95°C
Permeabilized with 0.1% Triton X100 in TBS

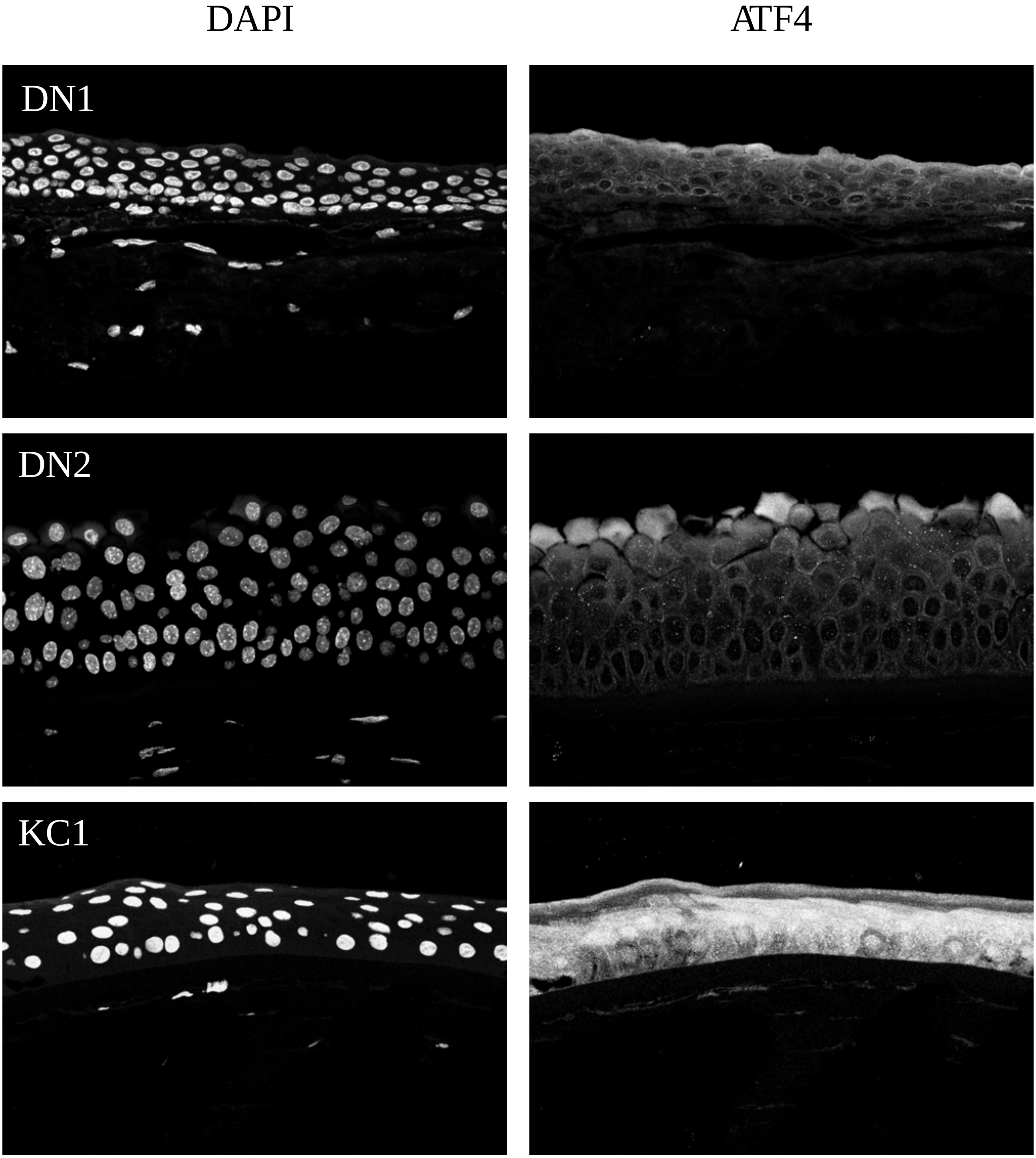
DAPI
ATF4
DN1
DN2
KC1

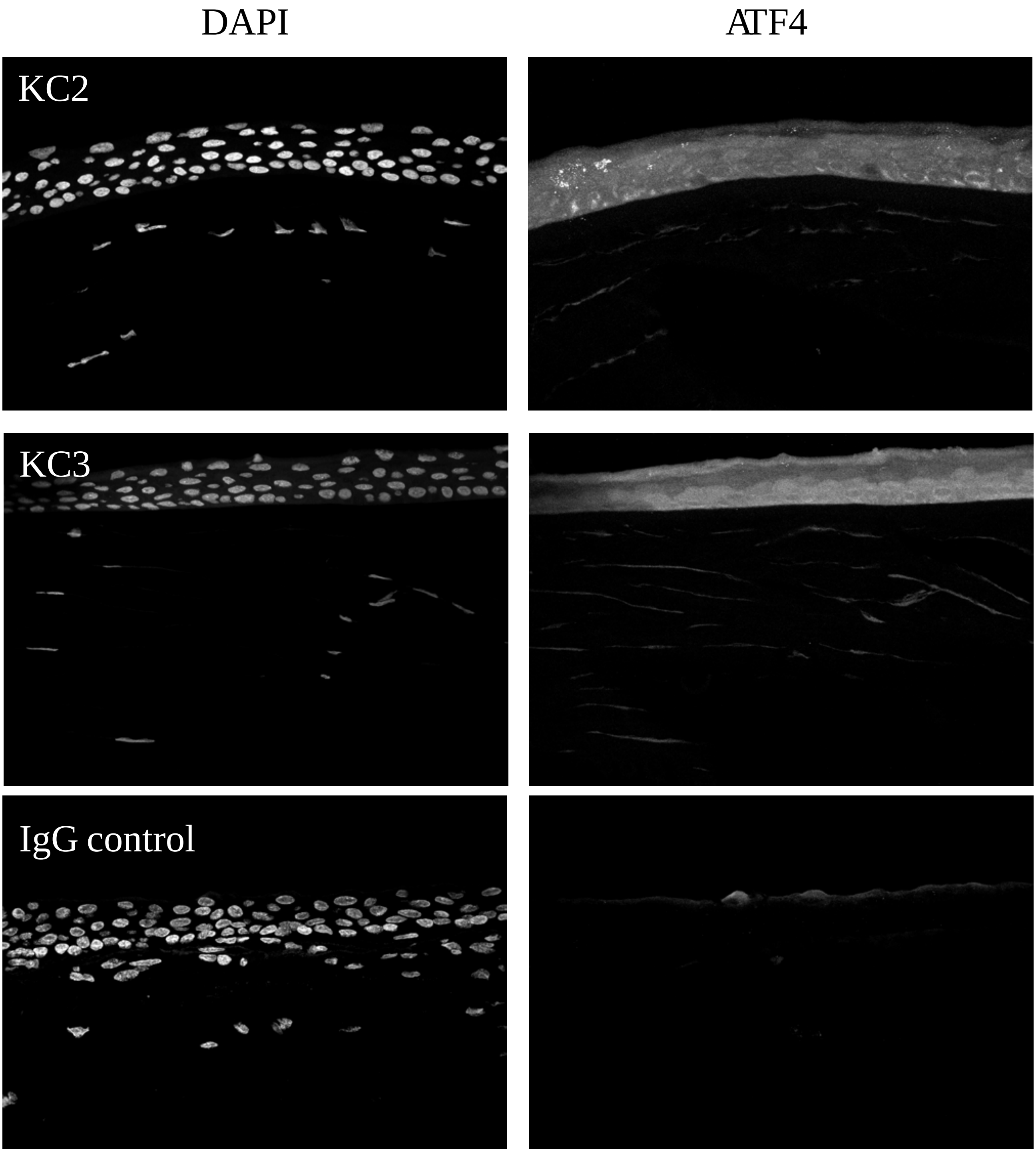

ATF4, Abcam ab184909. 1:500 in TBS 5% BSA, overnight 4°C.
Tris-EDTA pH 8.0 antigen retrieval, 20mins 95°C
Permeabilized with 0.1% Triton X100 in TBS
